# Association of deep tiny flow voids with prognosis of acute middle cerebral artery atherosclerotic occlusion

**DOI:** 10.3389/fnhum.2025.1578853

**Published:** 2025-04-03

**Authors:** Man-Qiu Ding, Wei-Zhuang Yuan, Zi-Jue Wang, Yue-Lun Zhang, Ming-Li Li, Yan Xu, Wei-Hai Xu

**Affiliations:** ^1^Department of Neurology, State Key Laboratory of Complex Severe and Rare Diseases, Peking Union Medical College Hospital, Chinese Academy of Medical Sciences and Peking Union Medical College, Beijing, China; ^2^Institute of Clinical Medicine, Peking Union Medical College Hospital, Chinese Academy of Medical Sciences and Peking Union Medical College, Beijing, China; ^3^Department of Radiology, Peking Union Medical College Hospital, Chinese Academy of Medical Sciences and Peking Union Medical College, Beijing, China

**Keywords:** deep tiny flow voids, middle cerebral artery, atherosclerosis, occlusion, prognosis

## Abstract

**Background:**

Deep tiny flow voids (DTFVs) have recently been identified as a novel form of collateral circulation linked to chronic steno-occlusive atherosclerotic middle cerebral artery (MCA) lesions, detectable via high-resolution magnetic resonance imaging (HR-MRI). To date, no study has focused on the presence and clinical significance of DTFVs in acute MCA atherosclerotic occlusion.

**Materials and methods:**

This retrospective study included patients with acute MCA atherosclerotic occlusion from two multicenter HR-MRI cohorts. The incidence of DTFVs and its association with baseline National Institute of Health Stroke Scale (NIHSS) scores, infarct volume, and the proportion of patients with a favorable 90-day clinical outcome defined as a modified Rankin Scale (mRS) ≤ 2 were analyzed.

**Results:**

Sixty-six patients (mean age 58.2 ± 9.2 years; 71.2% men) were included. The median time from stroke onset to image was 44.5 (27.3–67.0) hours. DTFVs were identified in 57.6% of patients with MCA atherosclerotic occlusion. After adjusting the potential confounders, DTFVs were significantly associated with lower baseline NIHSS scores (β, −3.68; 95% CI, −6.30, –1.07; *p* = 0.007), smaller infarct volume (β, −40.88; 95% CI, −70.15, −11.60; *p* = 0.007), and a higher proportion of patients with favorable 90-day clinical outcome (OR, 6.03; 95% CI, 1.39–26.19; *p* = 0.017).

**Conclusion:**

The presence of DTFVs was correlated with a favorable outcome in patients with acute MCA atherosclerotic occlusion. Improved recognition and awareness of this imaging marker of collaterals could help understand the varying infarct evolution seen in MCA occlusion and contribute to more individualized management and treatment.

## Introduction

Acute ischemic stroke due to middle cerebral artery (MCA) occlusion is common in stroke practice. Without reperfusion therapy, up to 87% of patients with proximal MCA occlusion and 47% with distal MCA occlusion have poor outcomes (Hernández-Pérez et al., 2014). Although intravenous thrombolysis within 9 h or endovascular treatment within 24 h from stroke onset can improve the prognosis (Liyis et al., 2023; Nogueira et al., 2018; Albers et al., 2018; Ma et al., 2019; Berge et al., 2021; Liu et al., 2023), a large number of patients do not present within these critical time windows (Tong et al., 2012; Yuan et al., 2023). Understanding the determinants of prognosis in such cases is essential for developing individualized treatment strategies and informing future clinical trials.

Collateral circulation is widely recognized as a key pathophysiological factor influencing the natural progression of ischemic brain injury (Lin et al., 2021; Lima et al., 2010). These collaterals primarily involve tertiary pathways, including the Circle of Willis, leptomeningeal vessels, and neovascularization (Leng and Leung, 2023). In the context of MCA occlusion, leptomeningeal collaterals have been strongly associated with favorable clinical outcomes (Lima et al., 2010; Madelung et al., 2018; Singer et al., 2015), whereas the role of the Circle of Willis remains controversial due to significant anatomical variability among individuals (Westphal et al., 2021; Sadeh-Gonik et al., 2024). Research on neovascularization, however, has been limited by the resolution constraints of conventional imaging techniques (Leng and Leung, 2023).

Recently, deep tiny flow voids (DTFVs) have been identified as a novel form of collateral circulation linked to steno-occlusive atherosclerotic MCA lesions, detectable via high-resolution magnetic resonance imaging (HR-MRI) (Xu et al., 2016; Xu et al., 2014). The prevalence of DTFVs increases with the severity of MCA stenosis and is higher in asymptomatic compared to symptomatic MCA occlusion cases (Xu et al., 2016). They were hypothesized to originate from new vessel network formation in response to chronic cerebral ischemia (Xu et al., 2016; Cho et al., 2013). Despite these insights, the incidence of DTFVs in acute MCA atherosclerotic occlusion and their impact on prognosis remain unexplored.

This study aims to investigate the presence and clinical significance of DTFVs in patients with acute ischemic stroke caused by MCA atherosclerotic occlusion. By doing so, we hope to shed light on their potential role in influencing outcomes and guiding therapeutic interventions.

## Methods

### Study design and participants

We conducted this study based on two multicenter cohort studies with similar designs: the Stroke Imaging Package Study (SIPS) (Zhang et al., 2023) and the Stroke Imaging Package Study of Intracranial Atherosclerosis (SIPS-ICAS) (Lin et al., 2020). The purpose of these two cohort studies was to evaluate the clinical value of HR-MRI in acute ischemic stroke, and the details of the study design were published elsewhere (Zhang et al., 2023; Lin et al., 2020). These two studies included patients who experienced their first-ever acute ischemic stroke within 7 days of symptom onset (Zhang et al., 2023; Lin et al., 2020). Age, sex, risk factors, onset to HR-MRI time, baseline NIHSS, reperfusion therapy, other treatment, mRS scores at 90 days and other information were recorded in detail. Patients with acute ischemic stroke due to MCA (including M1 or M2 segments) atherosclerotic occlusion, complete clinical data, and good image quality were selected for this study. Both SIPS and SIPS-ICAS were approved by the institutional review board at Peking Union Medical College Hospital, Chinese Academy of Medical Sciences (JS872 and JS-1699), and the local participating centers’ ethics board. The patients enrolled in both studies provided written informed consent.

Patients were excluded if they had any of the following characteristics: (1) coexistent with ipsilateral extracranial or intracranial internal carotid artery stenosis (≥ 50%), as assessed by MRA, Doppler ultrasonography, or CTA; (2) evidence of cardioembolism, including atrial fibrillation or flutter, left atrial thrombus, prosthetic valve, severe mitral stenosis, concomitant acute myocardial infarction, congestive heart failure, infective endocarditis, and/or sick-sinus syndrome as assessed by electrocardiogram or echocardiography; (3) evidence of vasculitis or arterial dissection, diagnosed by clinical evaluation, laboratory work, and vascular imaging; (4) Patients with undetermined etiology; (5) poor image quality.

### Image analysis

Both SIPS and SIPS-ICAS used the same imaging protocol, including conventional MRI (T1-weighted imaging, T2-weighted imaging, T2-weighted fluid attenuation inversion recovery imaging, diffusion-weighted imaging, three-dimensional time-of-flight magnetic resonance angiography, and susceptibility-weighted imaging or T2*-weighted imaging) and 3D T1-weighted HR-MRI (Zhang et al., 2023; Lin et al., 2020). Arterial spin labeling MRI, two-dimensional T2-weighted HR-MRI of intracranial arteries (bilateral middle cerebral arteries and basilar artery), and contrast-enhanced 3D T1-weighted HR-MRI were optional (Zhang et al., 2023; Lin et al., 2020).

**Figure 1:**
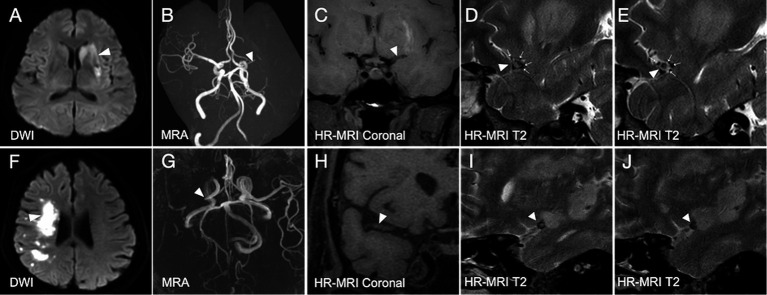
Illustrated cases of acute ischemic stroke due to middle cerebral artery occlusion with and without deep tiny flow voids. **(A–E)** In a patient with acute ischemic stroke (white arrowhead, **A**) due to the occlusive MCA (white arrowheads, **B–E**), DTFVs are revealed (white arrows, **D,E**). The 90-day mRS score for this patient is 0. **(F–J)** In another patient with acute ischemic stroke (white arrowhead, **F**) due to the occlusive MCA (white arrowheads, **G–J**), no DTFVs are revealed **(I,J)**. The 90-day mRS score for this patient is 3. DTFVs, deep tiny flow voids; MCA, middle cerebral artery; mRS, modified Rankin Scale.

As described previously, DTFVs were defined as three or more flow voids along the affected MCA on at least two consecutive T2-weighted image slices on HR-MRI (Figure 1) (Xu et al., 2016; Xu et al., 2014). Infarct volume was calculated using a self-developed algorithm based on the MATLAB platform. The total infarct volume for each patient was determined by multiplying the sum of the lesion areas in each slice by the slice thickness. For each slice containing infarct lesions, the radiologist selected a seed point within each lesion. The lesion was then segmented using a region-growing algorithm (Zhang et al., 2023; Adams and Bischof, 1994; Yuan et al., 2024).

Image analysis was performed using commercial software (Osirix MD, v.9.02). One experienced reader (Dr. Ding), blinded to the clinical information, interpreted the cross-sectional image slices of the bilateral MCA on HR-MRI. Dr. Ding and another experienced reader (Dr. Yuan) independently measured the DTFVs around occlusive MCA of the initial 20 consecutive patients two months later to assess interobserver and intraobserver variability. The intra-observer (κ = 0.89, *p* < 0.001) and inter-observer (κ = 0.80, *p* < 0.001) reproducibility of DTFVs measurements demonstrated substantial agreement.

### Definitions and outcomes

The disability at baseline and day 90 was evaluated by the National Institute of Health Stroke Scale (NIHSS) and modified Rankin Scale (mRS), respectively. A favorable 90-day clinical outcome was defined as a mRS score ≤ 2. The study outcomes included the incidence of DTFVs and the association of DTFVs with the baseline NIHSS, infarct volume, and the proportion of patients with a favorable 90-day clinical outcome.

### Statistical analysis

Continuous variables were described as mean (standard deviation [SD]) or median (interquartile range [IQR]), and categorical variables were presented as the frequency (percentage). We used Cohen’s ĸ coefficient to assess both interobserver and intraobserver agreement for DTFVs. Comparison between groups was performed using parametric (t-test or χ2 squared) or nonparametric (Wilcoxon or Fisher’s exact) tests. To adjust the potential confounders, including age, sex, hypertension, diabetes, hyperlipidemia, smoking, infarct volume, baseline NIHSS, and reperfusion therapy, we used linear or logistic regression analysis to investigate the association of DTFVs with baseline NIHSS, infarct volume, and the proportion of patients with a favorable 90-day clinical outcome. First, a univariate model was applied, including potential confounders, and the results were expressed in the regression coefficient (*β*) or odds ratio (OR) and 95% confidence interval (CI). All variables with *p* < 0.1 in the univariate analysis were included in the multivariate analysis. Variables with *p* < 0.05 were maintained in the analysis. A two-tailed *p*-value of less than 0.05 was considered significant for all statistical tests. All data analysis was conducted using SPSS Version 26.0 (IBM, Armonk, NY, United States).

## Results

**Figure 2:**
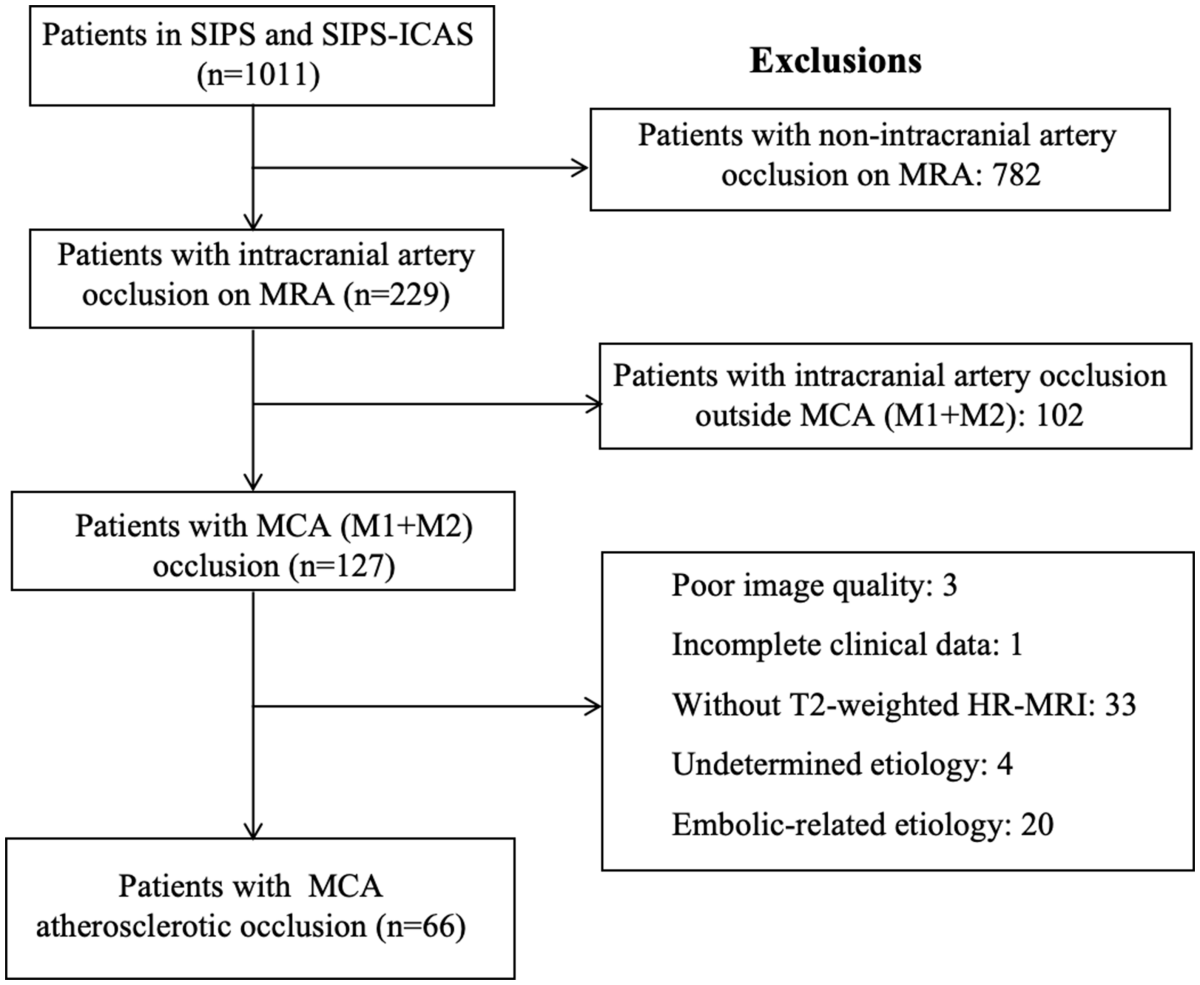
Flowchart of included/excluded patients. This flowchart presents the process of screening patients for enrollment from SIPS and SIPS-ICAS. MCA, middle cerebral artery; MRA, magnetic resonance angiography; SIPS, Stroke Imaging Package Study; SIPS-ICAS, Stroke Imaging Package Study of Intracranial Atherosclerosis.

Among 1,011 patients with acute ischemic stroke in two cohort studies, MCA (including M1 and M2) occlusion was observed in 127 patients based on the result of MRA. After excluding 20 patients with embolic-related occlusion, 4 patients with undetermined etiology, 33 patients without T2-weighted HR-MRI, 3 patients with poor image quality, and 1 with incomplete clinical data, 66 patients were enrolled for analysis, including 46 from SIPS and 20 from SIPS-ICAS (Figure 2). The mean age was 58.2 years (standard deviation [SD], 9.2 years), and 47 patients (71.2%) were male. The median time from symptom onset to imaging was 44.5 h (IQR, 27.3–67.0 h). The characteristics of this cohort are presented in Table 1.

**Table 1:** Characteristics of patients with or without DTFVs.

	Overall (*n* = 66)	With DTFVs (*n* = 38)	Without DTFVs (*n* = 28)	*P* Value
Age, mean (SD), y	58.2 (9.2)	57.8 (9.0)	58.6 (9.6)	0.73
Sex, male, *n* (%)	47 (71.2)	28 (73.7)	19 (67.9)	0.61
Risk factors, *n* (%)				
Hypertension	42 (63.6)	24 (63.2)	18 (64.3)	0.93
Diabetes	20 (30.3)	14 (36.8)	6 (21.4)	0.18
Hyperlipidemia	17 (25.8)	9 (23.7)	8 (28.6)	0.65
Current or previous smoking	32 (48.5)	22 (57.9)	10 (35.7)	0.08
Treatment, *n* (%)				
Reperfusion therapy	12 (18.2)	4 (10.5)	8 (28.6)	0.104
Antiplatelet	62 (93.9)	37 (97.4)	25 (89.3)	0.17
Lipid-lowering therapy	61 (92.4)	35 (92.1)	26 (92.9)	0.91
Onset to HR-MRI time, median (IQR), hours	44.5 [27.3–67.0]	38.0 [27.0–63.3]	48.0 [26.6–70.8]	0.40
Infarct volume, median (IQR), cm^3^	7.9 [3.4–22.9]	4.9 [2.6–10.4]	20.9 [8.2–75.2]	<0.001
Baseline NIHSS, median (IQR)	7.5 [2.8–13.0]	4.0 [1.0–8.0]	11.5 [7.3–14.8]	<0.001
Favorable outcomes at 90 days, *n* (%)	43 (65.2)	33 (94.3)	10 (35.7)	<0.001

HR-MRI, high-resolution magnetic resonance imaging; DTFVs, deep tiny flow voids; NIHSS, National Institute of Health Stroke Scale.

### Characteristics of patients with or without DTFVs

**Figure 3:**
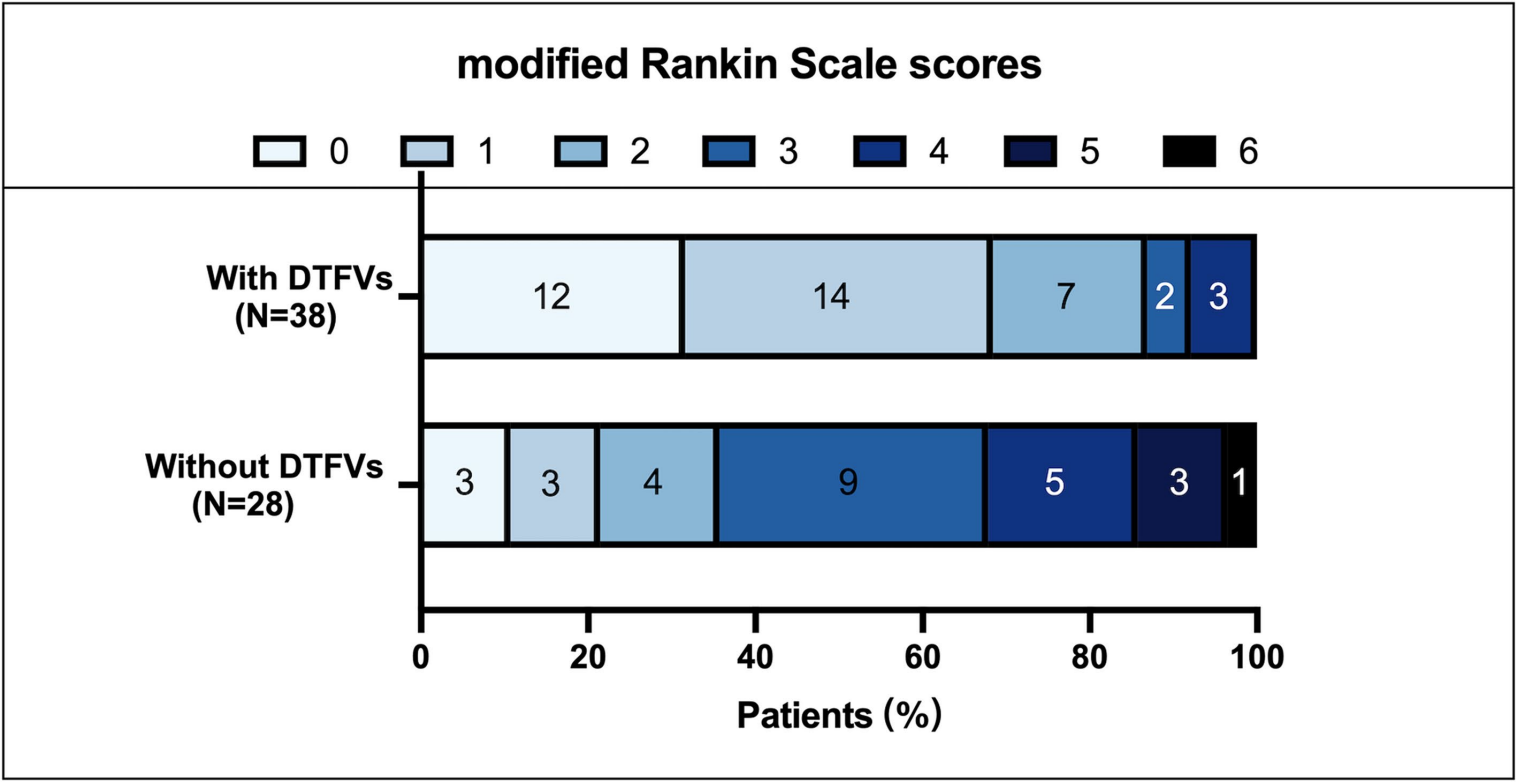
Distribution of 90-day modified Rankin scale scores in patients with and without DTFVs.

Among these 66 patients, 38 (57.6%) had DTFVs (Table 1). Table 1 shows the characteristics of patients with and without DTFVs. There are no significant differences between demographic characteristics, risk factors, onset to HR-MRI time, and all treatments between the two groups. Compared with patients without DTFVs, the patients with DTFVs had much lower NIHSS scores (4.0 [1.0–8.0] vs. 11.5 [7.3–14.8], *p* < 0.001), smaller infarct volumes (4.9 [2.6–10.4] cm^3^ vs. 20.9 [8.2–75.2] cm^3^, *p* < 0.001), and higher percentage patients having favorable clinical outcomes at day 90 (94.3% vs. 35.7%, *p* < 0.001) (Table 1; Figure 3).

### Associations of DTFVs with clinical outcomes

We screened for factors associated with smaller infarct volume, lower baseline NIHSS score, and favorable outcome on day 90 by univariate regression analysis, respectively, in patients with acute MCA atherosclerotic occlusion. Multivariate regression analyses were performed for variables with *p* < 0.1 in univariate regression analyses. After adjusting for age and smoking, DTFVs (β, −40.88; 95% CI, −70.15, −11.60; *p* = 0.007) were significantly associated with smaller infarct volume. After adjusting for hyperlipidemia and infarct volume, DTFVs (β, −3.68; 95% CI, −6.30, −1.07; *p* = 0.007) were significantly associated with lower baseline NIHSS score. After adjusting for smoking, infarct volume, and NIHSS score, DTFVs (OR, 6.03; 95% CI, 1.39, 26.19; *p* = 0.017) were significantly associated with a favorable outcome on day 90 (Table 2). Overall, DTFVs were significantly associated with smaller infarct volume, lower baseline NIHSS score, and a favorable outcome on day 90.

**Table 2:** Univariate and multivariate analyses of the association between DTFVs and clinical outcomes.

	Univariate analysis		Multivariate analysis	
Infarct volume	β (95% CI)	*P* value	β (95% CI)	*P* value
DTFVs	−47.97 (−77.51, −18.43)	0.002	−40.88 (−70.15, −11.60)	0.007
Age	1.42 (−0.27, 3.11)	0.097	0.97 (−0.61, 2.56)	0.224
Sex	−6.64 (−41.40, 28.12)	0.704		
Hypertension	25.84 (−6.27, 57.95)	0.113		
Diabetes	−25.74 (−59.41, 7.94)	0.132		
Hyperlipidemia	5.10 (−30.91, 41.11)	0.778		
Smoking	−41.34 (−71.13, −11.55)	0.007	−28.46 (−58.15, 1.22)	0.06
MCA-M1 occlusion	−31.44 (−79.07, −16.20)	0.192		
NIHSS scores	β (95% CI)	*P* value	β (95% CI)	*P* value
DTFVs	−5.29 (−7.85, −2.73)	<0.001	−3.68 (−6.30, −1.07)	0.007
Age	0.03 (−0.12, 0.19)	0.672		
Sex	−0.86 (−4.00, 2.28)	0.585		
Hypertension	0.23 (−2.73, 3.18)	0.879		
Diabetes	−2.89 (−5.90, 0.13)	0.060	−1.49 (−4.14, 1.17)	0.267
Hyperlipidemia	0.99 (−2.26, 4.23)	0.545		
Smoking	−2.24 (−5.03, 0.55)	0.114		
Infarct volume	0.04 (0.02, 0.06)	<0.001	0.03 (0.01, 0.05)	0.007
MCA-M1 occlusion	−2.91 (−7.21, 1.38)	0.180		
Favorable outcome on day 90	OR (95% CI)	*P* value	OR (95% CI)	*P* value
DTFVs	11.88 (3.52–40.14)	<0.001	6.03 (1.39, 26.19)	0.017
Age	0.98 (0.92–1.03)	0.371		
Sex	0.47 (0.16–1.41)	0.179		
Hypertension	0.90 (0.31–2.59)	0.845		
Diabetes	1.93 (0.60–6.23)	0.272		
Hyperlipidemia	0.50 (0.16–1.54)	0.224		
Smoking	1.80 (1.89–19.53)	0.002	5.55 (1.17, 26.37)	0.031
Infarct volume	0.98 (0.96–0.99)	0.007	0.99 (0.98, 1.01)	0.50
NIHSS score	0.78 (0.68–0.88)	<0.001	0.83 (0.72, 0.97)	0.017
Reperfusion therapy	0.46 (0.13–1.63)	0.230		
MCA-M1 occlusion	1.70 (0.32–9.21)	0.536		

DTFVs, deep tiny flow voids; NIHSS, National Institute of Health Stroke Scale; MCA, middle cerebral artery.

## Discussion

Our study investigated the presence and clinical significance of DTFVs in acute MCA atherosclerotic occlusion. We found that nearly half of the patients exhibited DTFVs. Compared to patients without DTFVs, those with DTFVs were more likely to present with lower NIHSS scores, smaller infarct volumes, and better 90-day prognoses. These findings highlight the potential role of DTFVs as a marker of favorable outcomes in acute MCA atherosclerotic occlusion.

We observed that patients with DTFVs exhibited lower baseline NIHSS scores and smaller infarct volumes. These findings align with our previous study, which reported a higher prevalence of DTFVs in patients with asymptomatic MCA occlusion compared to symptomatic cases (68% vs. 41.7%) (Adams and Bischof, 1994). This consistency suggests that DTFVs may serve as a protective factor in ischemic stroke. Similar to other collateral pathways, such as leptomeningeal vessels, DTFVs appear to mitigate the risk of stroke in large artery occlusive diseases and help reduce ischemic injury when a stroke occurs (Leng and Leung, 2023). Notably, our study demonstrated that DTFVs were significantly associated with favorable clinical outcomes, underscoring their potential value in guiding clinical decision-making. For instance, the presence of DTFVs in patients with acute MCA occlusion may indicate that aggressive or invasive therapies are less necessary. Conversely, patients without DTFVs may represent a subgroup that could benefit from more intensive interventions and should be prioritized in future clinical trials.

The underlying pathophysiology for DTFVs in acute MCA atherosclerotic occlusion remains incompletely understood. In our previous study, we found that the incidence of DTFVs increased with the degree of MCA stenosis, particularly in cases with 70% or greater stenosis or complete occlusion. This is consistent with the understanding that neovascularization—through mechanisms such as vasculogenesis, angiogenesis, and/or arteriogenesis—develops in response to chronic tissue hypoxia and injury (Ergul et al., 2014). This is also consistent with the phenomenon observed in coronary artery disease, that is, coronary lesion severity is the only independent pathogenetic variable related to collateral flow, which promotes enough collateral flow to prevent myocardial ischemia during coronary occlusion (Pohl et al., 2001). We hypothesize that patients without DTFVs may have had less severe MCA stenosis before acute occlusion compared to those with DTFVs. This hypothesis is supported by a similar phenomenon observed in coronary artery disease. In cases of mild to moderate coronary atherosclerosis, acute occlusion often leads to severe and potentially fatal myocardial infarction with large infarct areas due to the lack of well-developed collateral circulation (Hackett et al., 1988; Stone et al., 2023). In contrast, patients with severe coronary atherosclerosis may be asymptomatic or present with stable angina rather than acute infarction, as the chronic hypoxic stimulus promotes the formation of extensive collateral vessels, which can mitigate the effects of sudden occlusion (Pohl et al., 2001; Hackett et al., 1988; Stone et al., 2023). This parallel suggests that the absence of DTFVs in acute MCA occlusion may reflect insufficient pre-existing stenosis to trigger robust neovascularization, leaving the brain more vulnerable to ischemic injury when acute occlusion occurs. Conversely, patients with DTFVs may have had more severe and prolonged stenosis, leading to the development of protective collateral vessels that limit infarct size and improve outcomes. These insights highlight the potential role of DTFVs as a marker of pre-existing collateral development and their importance in understanding the variability of outcomes in acute MCA occlusion.

Our study has several limitations that should be acknowledged. First, as a retrospective study, the results may be influenced by selection bias, particularly given the characteristics of the included population. The patients were relatively young (mean age 58.2 years) and had small infarct volumes (median 7.9 cm^3^). The pre-designed extended MRI acquisition time may have further skewed the cohort toward those with mild to moderate symptoms, as patients with severe strokes may not have been stable enough to undergo prolonged imaging. To address this, future studies should aim to include a broader range of patients, encompassing a wider spectrum of ages, infarct sizes, and stroke severities. Second, HR-MRI is not routinely used in clinical practice, which may limit the immediate applicability of our findings. However, this limitation does not diminish the significance of our conclusions regarding the pathophysiology and clinical relevance of DTFVs. With advancements in accelerated MRI techniques and the integration of artificial intelligence in medical imaging (Pohl et al., 2001; Hackett et al., 1988), HR-MRI is poised to become more accessible and widely adopted in clinical settings. These technological developments hold promise for expanding the use of HR-MRI in routine stroke evaluation, enabling more precise identification of DTFVs and other collateral pathways. Third, the small sample size of our study may limit statistical power and increase bias risk. Nevertheless, our findings provide preliminary evidence suggesting that the presence of DTFVs is associated with favorable clinical outcomes in patients with acute ischemic stroke due to middle cerebral artery occlusion. Future multi-center studies with larger sample sizes are warranted to validate these observations and explore potential effect modifiers.

## Conclusion

In conclusion, our study demonstrates that the presence of DTFVs is associated with favorable outcomes in patients with acute ischemic stroke caused by MCA atherosclerotic occlusion. These findings highlight the potential role of DTFVs as an imaging marker of collateral circulation, which may help explain the variability in infarct evolution observed in MCA occlusion. Improved recognition and understanding of DTFVs could enhance our ability to predict patient outcomes and guide more individualized management and treatment strategies.

## Data Availability

The raw data supporting the conclusions of this article will be made available by the authors, without undue reservation.
