# Supplementary material for: Association of deep tiny flow voids with prognosis of acute middle cerebral artery atherosclerotic occlusion
Source: Front Hum Neurosci. 2025 Apr 3;19:1578853. doi: 10.3389/fnhum.2025.1578853 (PMC12003345; doi:10.3389/fnhum.2025.1578853)
Supplement: Supplementary file 1 [file Data_Sheet_1.pdf]

Supplementary Table 1 GE inventio MR 750

	HR-2D T2WI	HR-3D T1WI	SWI	DWI	SATISDATIO	T2WI	T1WI	T2* WI	3D TOF MRA
Sequentia nomen	FSE	CUBE a	Swan/ESWAN[†]	DWI	SATISDATIO	T2WI	T1WI	GR-T2*	3D TOF MRA
TR (ms)	2500-3600	500-600	22.4-77.8	NA	NA	NA	NA	NA	NA
TE (ms)	50-56	15-18	20-45	NA	NA	NA	NA	NA	NA
TI (ms)	NA	NA	NA	NA	NA	NA	NA	NA	NA
FA (^{0})	90-111	90-150	10-25	NA	NA	NA	NA	NA	NA
ST (mm)	1.5-2	0.8	1-4	NA	NA	NA	NA	NA	NA
Reconstruction ST(mm)	NA	0.4	NA	NA	NA	NA	NA	NA	NA
Hiatus (mm)	0.2-0.3	NA	NA	NA	NA	NA	NA	NA	NA
	HR-2D T2WI	HR-3D T1WI	SWI	DWI	SATISDATIO	T2WI	T1WI	T2* WI	3D TOF MRA
Numerus Verre	12-13	120-158	40-192	NA	NA	NA	NA	NA	NA
FOV (mm)	130	NA	NA	NA	NA	NA	NA	NA	NA
Acquisitionem propensionis	Obliquus sagittalis	Coronal Obliquus	Axial Obliquus	Axial R/L Obliquus	Axial A/P Obliquus	Axial A/P Obliquus	Axial A/P Obliquus	Axial Obliquus	Axial A/P Obliquus
Adeptio matrix	256* 256<br>384* 256	256* 256<br>288* 288<br>320* 256	320* 224<br>320* 256<br>384* 224<br>384*320<br>448*320	NA	NA	NA	NA	NA	NA
Reconstruction matrix	512*512	512*512	NA	NA	NA	NA	NA	NA	NA
Numerum signum acquisitionis	2-4	1	NA	NA	NA	NA	NA	NA	NA
Sensitivo ecode factor	NA	2（RL）	NA	NA	NA	NA	NA	NA	2(AP)
Oversampling factor	Ita	NA	NA	NA	NA	NA	NA	NA	1.8-3
	HR-2D T2WI	HR-3D T1WI	SWI	DWI	SATISDATIO	T2WI	T1WI	T2* WI	3D TOF MRA

**Echo agmine tandem**	24	24	NA	NA	NA	NA	NA	NA	NA
**Tempus acquisitionis (min: sec)**	1, 15-4, 54;	3:43-6:17	NA	NA	NA	NA	NA	NA	NA

Abbreviations: HR-2D T2WI, high-resolution two-dimensional T2-weighted imaging; HR-3D T1WI, high-resolution three-dimensional T1-weighted imaging; SWI, susceptibility-weighted imaging; DWI, diffusion-weighted imaging; FLAIR, fluid-attenuated inversion recovery imaging; T1WI, T1-weighted imaging; T2WI, T2-weighted imaging; T2WI, T2-weighted imaging; GRE, gradient echo; 3D TOF MRA, three-dimensional time-of-flight magnetic resonance angiography; FSE, fast spin echo; TR, repetition time; TE, echo time; TI, inversion time; FA, flip angle; ST, slice thickness; FOV, field of view; R/L, right/left; A/P, anterior/posterior; NA, not applicable.

Supplementary Table 2. Siemens scanner model

	HR-2D T2WI	HR-3D T1WI	SWI	DWI	SATISDATIO	T2WI	T1WI	T2* WI	3D TOF MRA
**Sequentia nomen**	FSE/*TSE	SPATIUM a	SWI/ Swan [a]	NA	NA	NA	NA	NA	NA
**TR (ms)**	2500-4000	500-900	27-28	NA	NA	NA	NA	NA	NA
**TE (ms)**	45-70	11-22	20	NA	NA	NA	NA	NA	NA
**TI (ms)**	NA	NA	NA	NA	NA	NA	NA	NA	NA
**FA (^{0})**	120-150	120	15	NA	NA	NA	NA	NA	NA
**ST (mm)**	2.0	0.63-0.8	1.2-3	NA	NA	NA	NA	NA	NA
**Reconstruction ST(mm)**	NA	0.3-0.4	NA	NA	NA	NA	NA	NA	NA
**Hiatus (mm)**	0-0.2	NA	NA	NA	NA	NA	NA	NA	NA
**Numerus Verre**	9-22	60-192	40-120	NA	NA	NA	NA	NA	NA
**FOV (mm)**	130-140	160-215	NA	NA	NA	NA	NA	NA	NA
**Acquisitionem propensionis**	Obliquus sagittalis R >> L	Coronal R >> L	Axial R >> L	Axial A >> P	Axial R >> L	Axial R >> L	Axial Sagittal A >> P	Axial	Axial R >> L
	**HR-2D T2WI**	**HR-3D T1WI**	**SWI**	**DWI**	**SATISDATIO**	**T2WI**	**T1WI**	**T2* WI**	**3D TOF MRA**
**Adeptio matrix**	256* 256<br>320*320	256* 256<br>256* 270	256* 223<br>256* 243<br>256* 256<br>320*320<br>320*240	NA	NA	NA	NA	NA	NA
**Reconstruction matrix**	512*512	512*512	NA	NA	NA	NA	NA	NA	NA
**Numerum signum acquisitionis**	4-5	1-2	NA	NA	NA	NA	NA	NA	NA
**Oversampling factor**	Ita	NA	NA	NA	NA	NA	NA	NA	NA

**Echo agmine tandem**	14-32	19-71	NA	NA	NA	NA	NA	NA	NA
**Tempus acquisitionis (min: sec)**	2:40-7:23;	4, 47-7:51;	NA	NA	NA	NA	NA	NA	NA

Abbreviations: HR-2D T2WI, high-resolution two-dimensional T2-weighted imaging; HR-3D T1WI, high-resolution three-dimensional T1-weighted imaging; SWI, susceptibility-weighted imaging; DWI, diffusion-weighted imaging; FLAIR, fluid-attenuated inversion recovery; T1WI, T1-weighted imaging; T2WI, T2-weighted imaging; T2WI, T2-weighted imaging; GRE, gradient echo; 3D TOF MRA, three-dimensional time-of-flight magnetic resonance angiography; FSE, fast spin echo; TR, repetition time; TE, echo time; TI, inversion time; FA, flip angle; ST, slice thickness; FOV, field of view; R/L, right/left; A/P, anterior/posterior; NA, not applicable.

Supplementary Table 3. Philips scanner model

	HR-2D T2WI	HR-3D T1WI	SWI	DWI	SATISDATIO	T2WI	T1WI	T2* WI	3D TOF MRA
**Sequentia nomen**	FSE/TSE/FS T2W_DRIVE [a]	T1W VISTA [a]	Swan [a] / VEN_BOLD_HR[a]	NA	NA	NA	NA	NA	NA
**TR (ms)**	2500-3400	400-800	14-19	NA	NA	NA	NA	NA	NA
**TE (ms)**	55-60	18-19	20-27	NA	NA	NA	NA	NA	NA
**TI (ms)**	NA	NA	NA	NA	NA	NA	NA	NA	NA
**FA (^{0})**	90-120	90	10-11	NA	NA	NA	NA	NA	NA
**ST (mm)**	2	0.8	1	NA	NA	NA	NA	NA	NA
**Reconstruction ST(mm)**	2	0.4	NA	NA	NA	NA	NA	NA	NA
**Hiatus (mm)**	0.3-1	NA	NA	NA	NA	NA	NA	NA	NA
**Numerus scalpere**	12-28	130-140	120-240	NA	NA	NA	NA	NA	NA
**FOV (mm)**	130-200	180-200	NA	NA	NA	NA	NA	NA	NA
**Acquisitionem propensionis**	Obliquus sagittalis A >> P	Coronal R >> L	Axial R >> L	Axial A >> P	Axial R >> L	Axial R >> L A >> P	Axial R >> L	Axial R >> L	Axial R >> L
	**HR-2D T2WI**	**HR-3D T1WI**	**SWI**	**DWI**	**SATISDATIO**	**T2WI**	**T1WI**	**T2* WI**	**3D TOF MRA**
**Adeptio matrix**	260*201 260*248 360* 360 360* 398	240*236 316*316 316*317 332*301	220* 180 236* 205 276* 197 244* 200	NA	NA	NA	NA	NA	NA
**Reconstruction matrix**	800*800	480*480	NA	NA	NA	NA	NA	NA	NA
**Numerum signum acquisitionis**	2-4	1-2	NA	NA	NA	NA	NA	NA	NA
**Sensitivo ecode factor**	1.5	1.8-2	NA	NA	NA	NA	NA	NA	NA

**Oversampling factor**	NA	NA	NA	NA	NA	NA	NA	NA	NA
**Echo agmine tandem**	10-19	24-30	NA	NA	NA	NA	NA	NA	NA
**Tempus acquisitionis (min: sec)**	3:36-5, 33 .;	VI, 5-7: 57»	NA	NA	NA	NA	NA	NA	NA

Abbreviations: HR-2D T2WI, high-resolution two-dimensional T2-weighted imaging; HR-3D T1WI, high-resolution three-dimensional T1-weighted imaging; SWI, susceptibility-weighted imaging; DWI, diffusion-weighted imaging; FLAIR, fluid-attenuated inversion recovery; T1WI, T1-weighted imaging; T2WI, T2-weighted imaging; T2WI, T2-weighted imaging; GRE, gradient echo; 3D TOF MRA, three-dimensional time-of-flight magnetic resonance angiography; FSE, fast spin echo; TR, repetition time; TE, echo time; TI, inversion time; FA, flip angle; ST, slice thickness; FOV, field of view; R/L, right/left; A/P, anterior/posterior; NA, not applicable.
