# Supplementary material for: Association of deep tiny flow voids with prognosis of acute middle cerebral artery atherosclerotic occlusion
Source: Front Hum Neurosci. 2025 Apr 3;19:1578853. doi: 10.3389/fnhum.2025.1578853 (PMC12003345; doi:10.3389/fnhum.2025.1578853)
Supplement: Supplementary file 2 [file Data_Sheet_2.pdf]

Supplementary Table 4: Multivariate analyses of the association between DTFVs and clinical outcomes (Model 2).

	Univariate analysis		Multivariate analysis	
**Infarct volume**	**β (95% CI)**	**P Value**	**β (95% CI)**	**P Value**
DTFVs	-47.97 (-77.51, -18.43)	0.002	-39.26 (-69.38, -9.13)	0.011
Age	1.42 (-0.27, 3.11)	0.097	0.94 (-0.66, 2.54)	0.246
Sex	-6.64 (-41.40, 28.12)	0.704		
Hypertension	25.84 (-6.27, 57.95)	0.113		
Diabetes	-25.74 (-59.41, 7.94)	0.132		
Hyperlipidemia	5.10 (-30.91, 41.11)	0.778		
Smoking	-41.34 (-71.13, -11.55)	0.007	-27.88 (-57.84, 2.08)	0.068
Reperfusion therapy	29.19 (-11.01, 69.29)	0.512	9.83 (-28.36, 48.03)	0.609
MCA-M1 occlusion	-31.44 (-79.07, -16.20)	0.192		
**NIHSS scores**	**β (95% CI)**	**P Value**	**β (95% CI)**	**P Value**
DTFVs	-5.29 (-7.85, -2.73)	<0.001	-3.68 (-6.30, -1.07)	0.007
Age	0.03 (-0.12, 0.19)	0.672		
Sex	-0.86 (-4.00, 2.28)	0.585		
Hypertension	0.23 (-2.73, 3.18)	0.879		
Diabetes	-2.89 (-5.90, 0.13)	0.060	-1.49 (-4.14, 1.17)	0.267
Hyperlipidemia	0.99 (-2.26, 4.23)	0.545		
Smoking	-2.24 (-5.03, 0.55)	0.114		
Infarct volume	0.04 (0.02, 0.06)	<0.001	0.03 (0.01, 0.05)	0.007
MCA-M1 occlusion	-2.91 (-7.21, 1.38)	0.180		
**Favorable outcome on day 90**	**OR (95% CI)**	**P Value**	**OR (95% CI)**	**P Value**
DTFVs	11.88 (3.52-40.14)	<0.001	6.03 (1.39, 26.19)	0.017
Age	0.98 (0.92-1.03)	0.371		
Sex	0.47 (0.16-1.41)	0.179		
Hypertension	0.90 (0.31-2.59)	0.845		

Diabetes	1.93 (0.60-6.23)	0.272		
Hyperlipidemia	0.50 (0.16-1.54)	0.224		
Smoking	1.80 (1.89-19.53)	0.002	6.00 (1.22, 29.42)	0.027
Infarct volume	0.98 (0.96-0.99)	0.007	0.99 (0.98, 1.01)	0.427
NIHSS score	0.78 (0.68-0.88)	<0.001	0.82 (0.70, 0.96)	0.012
Reperfusion therapy	0.46 (0.13-1.63)	0.230	2.52 (0.36, 17.78)	0.354
MCA-M1 occlusion	1.70 (0.32-9.21)	0.536		

We incorporated variables with a $p < 0.1$ from the univariate regression analysis, as well as reperfusion therapy, into the multivariate regression model.

Abbreviations: DTFVs, deep tiny flow voids; NIHSS, National Institute of Health Stroke Scale; MCA, middle cerebral artery.
